# Dysphagia Assessments as Criteria in the ‘Decision-Making Process’ for Percutaneous Endoscopic Gastrostomy Placement in People with Amyotrophic Lateral Sclerosis: A Systematic Review

**DOI:** 10.1007/s00455-024-10686-2

**Published:** 2024-03-22

**Authors:** Ermioni Kotsia, Elizabeth Chroni, Anna Alexandropoulou, Claire Mills, Dimitra Veltsista, Zinovia Maria Kefalopoulou, Emilia Michou

**Affiliations:** 1 School of Medicine, Unit of Neuromuscular Diseases University Hospital of Patras, University of Patras, Patras, Greece; 2 Department of Neurology, School of Medicine, University of Patras, Patras, Greece; 3 School of Medicine, National and Kapodistrian University of Athens, Athens, Greece; 4 Speech and Language Therapy Department, Leeds Teaching Hospitals NHS Trust, Leeds, UK; 5 Leeds Institute of Health Sciences, University of Leeds, Leeds, UK; 6 Department of Speech and Language Therapy, School of Health Rehabilitation Sciences, University of Patras, Patras, Greece; 7 Centre for Gastrointestinal Sciences, Faculty of Biology, Medicine and Health, University of Manchester, Manchester Academic Health Sciences Centre (MAHSC), Manchester, UK

**Keywords:** Motor neuron disease, Screening, Deglutition, Swallowing, Tube feeding, Food modification

## Abstract

To review the assessment methods of dysphagia as a criterion for the decision-making process for Percutaneous Endoscopic Gastrostomy (PEG) placement in patients with Amyotrophic Lateral Sclerosis (ALS). Systematic review. A search was conducted in three databases (EMBASE, CINAHL, PUBMED) in December 2022 and updated in July 2023. Two reviewers independently screened, selected, and extracted data. Study quality was appraised using the Joanna Briggs Institute Critical Appraisal Tools. Systematic review registration number in the International Prospective Register of Systematic Reviews (PROSPERO): CRD42022385461. The searches identified 240 records. The 10 eligible studies included 2 case reports, 4 retrospective studies, 3 prospective studies, and 1 cohort observational study. Study quality was low, with most studies having moderate to high risk of bias. Dysphagia is a common criterion for decision-making. Dysphagia assessment is usually in the form of either self-reports, objective instrumental assessments, or both. Dysphagia is a common criterion for the decision-making process, yet is missing in clinical guidelines. Establishing the optimal means of dysphagia assessment is important for timely decision-making procedures, so that life-threatening consequences of dysphagia are minimized.

**Supplementary Information:**

The online version contains supplementary material available at 10.1007/s00455-024-10686-2.

## Introduction

Amyotrophic Lateral Sclerosis (ALS) is a progressive neurodegenerative disorder characterized by loss of the upper and lower motor neurons for spinal and musculature [1]. Degeneration of motor neurons leads to weakness of the striated muscles that control limb mobility, movement, respiration, speech, and swallowing [1]. Non-bulbar onset of the disease is diagnosed in about 70% of the cases, presenting with asymmetric painless weakness, cramps and atrophy in the arm and/or leg muscles, while the remaining 30% are diagnosed with bulbar onset of the disease, with prominent symptoms of dysarthria or/and dysphagia [1].

People living with ALS (PwALS) will inevitably develop dysphagia at some point during the disease. This typically occurs during the later stages of the disease; however, individuals who have a bulbar onset will likely experience swallowing impairments, and often severe dysphagia, much earlier [2, 3]. This is anticipated since the sensorimotor act of swallowing requires the activation of a diverse neuronal network, namely the bulbar motor neurons, cortical and subcortical areas connected via a fine-tuned corticobulbar network.

Dysphagia in ALS is related to tongue atrophy, dysfunction in palatopharyngeal closure, respiratory-swallow incoordination and weakness or incoordination of masticatory muscles impeding bolus manipulation and transfer [2, 3]. Dysphagia-related complications such as aspiration pneumonia, malnutrition, and dehydration increase the risk of death in patients with ALS [4]. Inadequate and/or unsafe food and fluid intake typically increase mortality rates and thus dysphagia acts as a key negative prognostic factor in ALS [4, 5]. When *per os* feeding becomes impossible or unsafe, nutrition and hydration are usually provided through Percutaneous Endoscopic Gastrostomy (PEG) [4, 6]. Placement of a PEG in PwALS likely prolongs survival. However, the ideal timing of PEG placement is not clearly determined by clinical evaluation. The guidelines for the management of ALS [7] recommend PEG when there is symptomatic dysphagia, when the weight loss exceeds 10% of the baseline value, and the forced vital capacity (FVC) decreases below 50% of the predicted level. Clinical guidelines can guide clinicians regarding the timing of PEG insertion, however most healthcare professionals find the issue of PEG insertion timing to be extremely challenging [8] because there is little evidence to support decision-making [9].

Generally, guidelines recommend that PEG is indicated for those with symptomatic dysphagia and associated weight loss [10, 11]. However, the term ‘symptomatic dysphagia’ in this population is vaguely defined and clinicians may use different screening or assessment tools to determine the presence and severity of dysphagia symptoms or the threshold of ‘symptomatic’ dysphagia. It is possible that these screening and assessment tools have variable psychometric properties and, therefore, they may not provide the most accurate information to the clinicians. Furthermore, little is known about how the results of any assessment procedures guide or play a role in ‘decision-making processes’ for altering the feeding route in PwALS or instigating PEG insertion. Here we systematically review the literature for the different assessment procedures used in the clinical settings to guide ‘decision-making’ procedures for PEG placements in PwALS.

## Methodology and Methods

A protocol for this systematic review was developed and registered with the International Prospective Register of Systematic Reviews (PROSPERO) (registration number: CRD42022385461). This systematic review was conducted and reported in accordance with the Preferred Reporting Items for Systematic Review and Meta-Analysis (PRISMA) reporting guidelines [12].

### Study Eligibility Criteria

The eligibility criteria for this review were designed according to the Population Intervention Comparators Outcomes Study (PICOS) framework. The *Population* included PwALS adult patients (≥ 18 years old) with dysphagia. We accepted studies with participants who had a diagnosis of definite, possible, or probable ALS, consistent with the El Escorial criteria [13] and Awaji criteria [14]. The *Intervention* was the dysphagia assessment. The *Comparison* in the literature was the clinical decision-making for PEG insertion. Studies with all *Outcome* measures were included. Qualitative and quantitative *Study* types were included, ranging from Randomized Control Trials, nonrandomized, observational and retrospective studies. Publications in English were included, published from 1975 to 2023.

### Search Strategy

In December 2022 the following databases were searched: Pubmed, Embase, and CINAHL Please see Supplemental Material Table S1 for search terms. The searches were repeated in the same databases on July 2023. Further relevant studies were sought by citation searching of the included studies. Studies were independently screened by two reviewers (E.K. and A.A.) to identify studies that met the inclusion criteria. Any disagreement was resolved through discussion between the two reviewers and, when necessary, with the wider review team. The reason for exclusion was documented.

### Data Extraction

Data extraction from included full-text articles consisted of author, year, country, title, aim, study population, setting, study design, outcome measures, and main findings. Two reviewers independently extracted data for all eligible studies. Any discordances between the completed extraction forms were identified and discussed. One additional table included information regarding the different dysphagia screening and assessment tools and specific information regarding the introduction of the assessment and screening, i.e., timing. All headings for the data extraction tables were developed and agreed upon by all reviewers before the extraction. These data extraction tables allowed findings from articles to be linked together, forming the basis of the results section of this review. Any differences during data extraction were resolved through discussion between the two reviewers and, where necessary, with the wider review team.

### Risk of Bias Assessment

The Joanna Briggs Institute (JBI) recommendations for levels of evidence were used to rate each study. Risk of Bias Assessment (RoB) was assessed for each study independently by the two reviewers using JBI Critical Appraisal checklists. The following JBI Critical Appraisal checklists were used: case reports, case series, cohort studies, and cross-sectional studies (https://jbi.global/critical-appraisal-tools). This included assessment of (where applicable): reporting bias, internal validity, external validity, measurement bias, selection bias, power, attrition bias, confounding bias, performance bias, and detection bias. There is no scoring system for these checklists. Any discrepancies in the RoB analysis were resolved through discussion and a consensus decision was made.

## Results

### Search Results

**Fig. 1:**
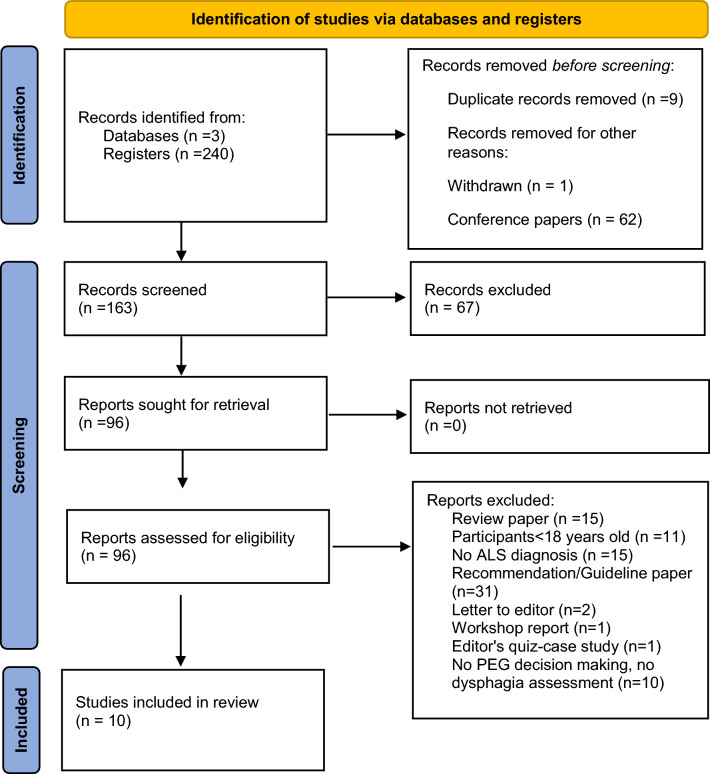
PRISMA flow diagram

Database searches identified 240 records. After duplicate removal, there were 230 records remaining. One study was withdrawn, and another was removed because it was not published in English. Citation searches did not identify any additional records. In total, 163 records were reviewed as shown on PRISMA flow diagram illustrating the selection process (Fig. 1). Of those, 96 papers were assessed for eligibility, of which 15 were review papers. The final review was conducted on 10 studies, published between 1996 and 2023.

### Study Results

#### Study Characteristics

The study characteristics are outlined in Table 1. Studies were predominantly prospective and retrospective, with two case report studies. A total of 846 patients were included in this review, 418 (49.4%) were female and 428 (50.6%) were men, with an age range of 20–80 and a sample size range of 1–193. The final review was conducted on 10 studies from the USA [15–17], Italy [18, 19], UK [20], Brazil [21], South Korea [22], Australia [23], and Lithuania [24]. Settings varied from specialty clinic service for MND [15, 18, 19, 23], to neurology departments [22, 24], university hospitals and tertiary-care referral centers [16, 17, 21].

**Table 1:** Study findings

Study	Study Design	Study setting	Gender	Age (± Standard Deviation)	Age at diagnosis	Time to PEG since Diagnosis	PEG insertion Criteria	Dysphagia Assessment
Strand et al. (1996)^15^	Prospective	Neuromuscular Clinic for Swallowing and Speech Disorders	66F, 74 M	Mean: 59.8 (± 11.3)	NR	NR	VC, neurologic examinations, Swallowing Scale of the ALSSS, examination of the structure and function of the oral articulatory, VFSS if needed	Swallowing Scale of ALSSS, VFSS if needed
Shaw et al. (2006)^16^	Retrospective	Single center institution	49F, 49 M	Mean: 61 (SD NR)	NR	19 months for RIG, 15 months for PEG, 11 months for NG	One or more of the following criteria: a BMI of < 18.5 kg/m^2^, weight loss of at least 10% from pre-illness weight, or dysphagia graded 6/10 on the ALSSS	Swallowing Scale of ALSSS
Luchesi et al. (2014)^17^	Prospective	University hospital	15F, 18 M (Bulbar onset ALS: 3F, 2 M), (Spinal onset ALS: 12F, 16 M)	Mean: 55.6 (± 8.15)	NR	Patients with bulbar onset ALS: 28.8 (± 19.3) months, Patients with spinal onset ALS: 48 (± 27.3) months	FOIS, FEES, patients were observed until the point in time at which they required tube feeding and were classified at FOIS level 3	FOIS, FEES
Fasano et al. (2017)^18^	Cohort Observational population-based, registry study	Neurology Departments	101F, 92 M	Mean: 68.98 (± 9.9)	NR	26.56 (± 18.8) months	Swallowing scale of ALSFRS-R score, respiratory function values, height and body weight, and need for procedures (NIV, IV)	Swallowing scale of ALSFRS
Suh et al (2019)^19^	Retrospective analysis of prospectively collected data	Neurology department	ALS Group: 20F, 21 M, Healthy Group: 8F, 12 M	ALS group mean: 65.14 (± 10.99) Healthy group mean: 40.45 (± 15.08)	NR	NR	HRM, VFSS, FOIS, > 5% weight loss in the previous month or > 10% weight loss in the previous 6 months, PFT assessing FVC, FEV1, and FEV1/FVC	HRM, VFSS, FOIS
Labra, et al (2020)^20^	Prospective, cross-sectional study	Multidisciplinary, specialty MND Service	13F, 20 M	Mean: 68	NR	on average 214 days after initial contact with the service (range 0–1365 days)	Gastrostomy need was identified during routine care, following deterioration in the patients’ swallowing, nutrition or breathing	ALSFRS-R, MASA, FOIS, SWAL-QoL
Tye et al (2021)^21^	Retrospective	Τertiary-care center	87F, 91 M	64.8 (± 14)	NR	NR	EAT-10, FEES, FOIS	EAT-10, FEES, FOIS
Mariani et al (2021)^22^	Retrospective	Rare Neuromuscular Disease Centre	58F, 50 M	65.75 (± 10.49)	NR	25.78 (± 16.08) months	FEES, score of PAS, PI in relation to dysphagia onset and PEG indication (according to ALSFRS score)	FEES, Score of PAS
Videnovic et al. (2022)^23^	Case report	Hospital, Sleep clinic	1 M	67	64	NR	VFSS with laryngeal penetration and silent aspiration	VFSS
Rugaitienė et al. (2022)^24^	Case report	Geriatric Department	1F	66	66	9 months	DHI, SWAL-QoL, SWAL-CARE, EAT-10, FEES, Score of PAS	DHI, SWAL-QoL, SWAL-CARE, EAT-10, FEES, Score of PAS

*NR* Not Reported; *VC* Vital Capacity; *SLSSS* ALS Severity Scale; *VFSS* Videofluoroscopic Swallowing Study; *BMI* Body Mass Index; *FOIS* Functional Oral Intake Scale; *FEES* Fiberoptic Endoscopic Evaluation of Swallowing; *ALSFRS* ALS Functional Rating Scale; *ALSFRS-R* ALS Functional Rating Scale–Revised; *NIV* Non-invasive ventilation; *IV* Invasive Ventilation; *HRM* High-Resolution Manometry; *PFT* Pulmonary Function Test; *FVC* Forced Vital Capacity; *FEV1* Forced Expiratory Volume in 1 s; *MASA* Mann Assessment of Swallowing Ability; *SWAL-QoL* Swallowing-Quality of Life Scale; *EAT-1O* Eating Assessment Tool-10; *PAS* Score of Penetration Aspiration Scale; *PI* Progression Index; *DHI* Dysphagia Handicap Index; *SWAL-CARE* Swallowing Quality of Care

#### Study Quality

According to the JBI recommendations for reviewing the study quality, levels of evidence were generally low (from level 4.d to level 3.c). All studies had a moderate to high level of bias, with RoB in multiple domains for most studies (Table 2). Bias was observed in the following domains: reporting bias (9/10 studies), internal validity (10/10 studies), external validity (5/10), selection bias (4/10), confounding bias (6/10), performance bias (9/10), and detection bias (8/10).

**Table 2:** This table presents the different outcome measures and cut offs utilized in the studies included in the systematic review

Objective Dysphagia Diagnosis	Study	Outcome Measurements	Cutoff
FEES	Luchesi et al. (2014)	Presence or absence of larynx penetration and/or aspiration	PAS: NR FOIS level III
	Mariani et al. (2021)	FEES: Presence or absence of penetration or aspiration	PAS ≥ 6
VFS	Suh et al. (2019)	Penetration/aspiration Laryngeal elevation and oral phase duration FOIS	PAS ≥ 6 Laryngeal elevation & oral phase duration: NR FOIS level I-III
	Videnovic et al. (2022)	Penetration and aspiration	NR
HRM	Suh et al. (2019)	Swallowing pressure along the velopharynx (VP), tongue base (TB), pre upper esophageal sphincter, lower pharynx, cricopharyngeus, minimal upper esophageal sphincter (UES) pressure, the area integral, rise time, duration of VP, TB, UES, nadir UES, the maximal pressure, minimal pressure, area integral to the pressure peak, and timing intervals between the variables	NR
Subjective Dysphagia Diagnosis	Study	Outcome Measurements	Cutoff
ALSSS	Shaw et al. (2006)	Swallowing ability	Score 6/10
ALSFRS-R	Fasano et al. (2017)	Gastrostomy need	Score of 1 for item 3 on the scale
Combo Dysphagia Diagnosis			
Study	Technique	Outcome Measurements	Cutoff
Strand et al. (1996)	ALSSS	Swallowing ability	NR
	VFSS	Swallowing function and safety	NR
Labra, et al. (2020)	ALSFRS-R	Overall function rating	NR
	MASA	Overall function score	NR
	SWAL-QoL	Quality of life measure / Swallowing ability	NR
	FOIS	Functional oral intake score	NR
Tye et al. (2021)	EAT-10	Participant perception of swallowing difficulties	NR
	FEES	Presence or absence of penetration/aspiration	NR
Rugaitienė et al. (2022)	EAT-10	Patient’s Symptom Report	NR
	DHI	Symptoms and QoL	NR
	SWAL-QoL	QoL measure / Swallowing ability	NR
	SWAL-CARE	Carers’ QoL and Swallowing	NR
	FEES	Swallowing function and safety	NR

*FEES* Fiberoptic Endoscopic Examination of Swallowing [41], *VFS* Videofluoroscop]y [42], *HRM* High resolution manometry [43], *PAS* Penetration aspiration score [44], *FOIS* Functional Outcome Intake Scale [25], *EAT-10* Eating Ability Test-10 [26], *ALSFRS–R* ALS Functional Rating Scale–Revised[45], *ALSSS* ALS Swallowing Severity Scale[46], *MASA* Mann Assessment of Swallowing ability [47], *SWAL-QOL* Swallowing Quality of life [27], *SWAL-CARE* Swallowing Quality of Care questionnaire [27], *DHI* Dysphagia Handicap Index [28], *NR* None reported

#### Outcome measures

The outcome measures of ‘PEG insertion’ decision used most frequently were progression of dysphagia in ALS patients, variables related to faster progression of dysphagia and PEG placement [15, 19, 21], comparison to manometric, pulmonary and Videofluoroscopic study (VFSS) variables and PEG insertion [22], survival after PEG placement and prognostic factors related to PEG [18, 20]. One study [23] had outcomes measures of gastrostomy uptake and the acceptance and rejection reasons for PEG insertion.

#### Decision making criteria for PEG

The criteria for alteration of feeding route differed depending on the methodology of each study. In five studies [16, 17, 19, 21, 24] the decision for PEG insertion was made purely on the basis of the patients’ swallowing difficulties. In two studies [18, 22] the decision for PEG insertion was influenced by three factors, including weight loss, dysphagia, and respiratory function. In one study [15], PEG insertion criteria were neurologic examination, dysphagia, measurements of Forced Vital Capacity (FVC) and the results from the examination of oral structure and articulatory mechanism. Clinicians in one study [20] decided for PEG insertion based on one or more of the following criteria: body mass index (BMI), weight loss or dysphagia. In one study [23], PEG placement was made following deterioration in the patient’s swallowing, nutrition or breathing.

#### Assessment of dysphagia as a part of the ‘decision-making process’

Table 3 presents the different dysphagia assessments presented in the literature as far as decision-making process for PEG is concerned. These included either (A) imaging techniques such as VFSS or Fiberoptic Endoscopic Evaluation of Swallowing (FEES), (b) Questionnaires such as the Functional Oral Intake Scale (FOIS) [25], EAT-10 [26] and (c) a combination of both objective imaging and self-reported questionnaires [15, 16, 23, 24]. Table 3 presents the different outcome measures used in each of the objective assessment or self-reported scale, including the cut-offs used.

**Table 3:** Risk of Bias assessment

	Study Joanna Briggs Institute Appraisal Tool														
Study	Level of Evidence, Study Design	No Bias	Bias	Unclear	Not applicable	Reporting Bias	Internal Validity	External Validity	Measurement Bias	Selection Bias	Power	Attrition Bias	Confounding Bias	Performance Bias	Detection Bias
Strand et al. (1996)^ƚ^	Level 4.c, Case series	5	5	0	0	−	−	+	+	−	−	+	+	−	−
Shaw et al. (2006)^ƚ^	Level 4.c, Case series	4	4	2	0	−	−	−	+	+	−	+	−	−	−
Luchesi et al. (2014)^ƚ^	Level 4.c, Case series	8	0	2	0	+	−	+	+	+	−	+	+	+	+
Fasano et al. (2017)^ƚ^	Level 4.c, Case series	4	6	0	0	−	−	+	+	+	+	+	−	−	−
Suh et al (2019)	Level 3.c, Cohort study with control group	6	2	3	0	−	−	+	+	+	−	+	+	−	−
Labra, et al (2020)	Level 4.b, Cross-sectional study	5	2	1	0	−	−	−	+	+	−	+	+	−	−
Tye et al (2021)^ƚ^	Level 4.c, Case series	6	2	2	0	−	−	+	+	−	+	+	−	−	−
Mariani et al (2021)	Level 3.c, Cohort study with control group	6	2	3	0	+	−	+	+	+	−	−	−	−	+
Videnovic et al. (2022)	Level 4.d, Case study	7	1	0	0	−	−	−	+	−	−	+	−	−	−
Rugaitienė et al. (2022)	Level 4.d, Case study	4	3	1	0	−	−	−	+	+	−	+	−	−	+

^†^The case series critical appraisal checklist was used for these studies as one does not exist for retrospective and prospective studies without control groups

−  = high risk of bias; +  = low risk of bias

In all studies, the assessment and management of patients were conducted by a multidisciplinary team. Only two studies [15, 23] reported that a speech and language therapist (SLT) was a member of the multidisciplinary team. Six [16–19, 21, 24] did not explicitly state that an SLT was a member of the multidisciplinary team, however, from the methodology used, we could positively assume an SLT’s involvement.

#### Whole-Body measures and respiratory function

Three studies [18, 20, 22] considered BMI and weight loss as a criterion for PEG insertion. Despite the aforementioned international guidelines concerning PEG insertion criteria [7], only four studies [15, 18, 22, 23] defined the need for PEG by the patient’s respiratory function, among other criteria. Forced vital capacity (FVC) was the common outcome measure in these four studies.

## Discussion

We conducted a systematic review to explore how dysphagia assessments are used to assist the ‘decision-making process’ for tube feeding in PwALS. Ten studies were reviewed in depth and were a mixture of prospective, retrospective, and case report studies. The level of evidence of the studies was judged as low and there was a high RoB in more than three domains for every study included in this systematic review.

Despite the heterogeneity observed among the studies, we can conclude that presence of swallowing impairments (dysphagia) is a common criterion for PEG insertion in PwALS. However, there is high heterogeneity in clinical practice and the dysphagia assessment tools as part of the ‘decision-making process’ for PEG placement in PwALS differ greatly across the studies. Thus, our results merit further discussion.

With advancing dysphagia, PEG insertion can provide long-term nutritional support, prevent starvation, malnutrition, dehydration, pneumonia, and hypoxia, the latter when PEG insertion is accompanied by non-invasive ventilation [27]. There is an increasing trend of evidence supporting a beneficial effect of PEG insertion on survival of PwALS [27]. However, some controversial findings support that PEG insertion seems to be associated with faster functional decline and greater mortality hazard [27] in patients with more advanced disease. Thus, PEG insertion has been identified as having beneficial or detrimental outcomes according to the patients’ disease stage. Identification of the relevant predictive prognostic factors is thought to be important in determining the optimal timing of PEG insertion [27]. However, the optimal timing of PEG placement is not clearly determined by clinical guidelines.

During the decision-making process, the ‘deterioration of respiratory and swallowing functionality’ as well as the weight loss are perceived as criteria for PEG insertion [7]. However, there is heterogeneity among studies specifically concerning the tools and measurements which determine the deterioration of the criteria above. Also, there is heterogeneity in the number of criteria that are taken into account. There are studies [16, 17, 19, 21, 24] that the decision-making for PEG insertion is based only on swallowing impairment; while others [15, 18, 20, 22, 23] collect information on two criteria or more. Therefore, it is important to examine the different assessment and screening procedures in detail.

As far as the functional scales are concerned, Rooney et al. reported that the ALSFRS-R is the most frequently used scale to assess swallowing function in PwALS [28]. However, ALSFRS-R has only three items related to bulbar symptoms, including swallowing, speech, and salivation, which cannot fully express the needs of clinical practice in PwALS [29]. Another questionnaire based on self-reports for dysphagia is EAT-10 [29]. Reports have shown that ALSFRS-R bulbar subscale and EAT-10 could effectively identify unsafe swallowing and aspiration in PwALS [29, 30]. Specifically, a recent study [29] reported that EAT-10 demonstrated good discriminant ability to accurately identify penetration and aspiration in PwALS (PAS ≥ 3) with a cut-off score of 3 (AUC: 0.77, sensitivity: 88%, specificity: 57%) and that EAT-10 demonstrated excellent accuracy at identifying aspirators (PAS ≥ 6) utilizing a cut-off score of 8 (AUC: 0.88, sensitivity: 86%, specificity: 72%). In addition, the ALSFRS-R bulbar subscale had a sensitivity and specificity of 71.4% and 79.6%, respectively. Of the two tools, it seems that the EAT-10 could distinguish dysphagia more effectively than ALSFRS-R bulbar subscale [29]. However, a systematic literature review reported that EAT-10 shows poor internal consistency, reliability, and content validity [31]. Adding to the matter in hand, a recent study revealed that the 3 oz water swallow test was an inadequate screening tool to detect aspiration (low sensitivity: 55,2% and moderate specificity: 71,7%) (Donohue et al., 2022).

Concerning the instrumental endoscopic dysphagia assessments that have been used in studies in our systematic review there is some information on their clinimetric properties. A study reported that FEES detected the presence of bolus aspiration as well as the progressive worsening of some swallowing parameters, such as premature spillage and post-swallowing residue, as judged by FEES correlated with the severity of the disease (assessed through specific scores such as the ALSFRS and the b-ALSFRS) [32].

Similarly, with HRM, a study [22] identified that HRM parameters are significantly specific for the feeding type and the possibility of oral feeding in ALS. In addition, HRM could predict pulmonary function in patients with ALS [22]. The cut-off value of HRM parameters may be used to decide the feeding type in patients with ALS [22]. Suh et al., reported that a cut-off value of low pharyngeal pressure of 183.10 mm Hg showed 60.0% sensitivity and 88.9% specificity for the full oral and limited oral intake and the cut-off value of minimal Upper Esophageal Sphincter pressure of 5.65 mm Hg, had 80.0% sensitivity and 75.0% specificity.

We note that the tools used to assess swallowing in PwALS have controversial sensitivities and specificities, while swallowing assessments such as FEES, VFSS, and HRM have not been sufficiently studied in PwALS. For this reason, the utilization and interpretation of the results of these tools and tests is left to the good clinical practice and judgment abilities of the SLT.

Importantly, it is well known that the important interplay between respiratory and swallowing function [33] unveils in PwALS. There is an association between decreased vital capacity and increased swallowing difficulties [15]. Pulmonary function tests can provide important and necessary information on the prognosis in ALS and can help in determining the timing for long-term mechanical ventilation and end-of-life planning [34]. Garand et al. reported that FVC% pre is useful clinical indicator of oral and pharyngeal swallowing impairment in PwALS (Garand et al., 2023). In order to perform the procedure of PEG a relative unaffected respiratory function is necessary [7]. Vital capacity (VC), maximum mid-expiratory flow rate (MMEFR), forced vital capacity (FVC), and forced expiratory volume in 1s (FEV1) are the common measures used in PwALS [34]. Other recommendations include nocturnal pulse oximetry, maximal inspiratory pressure (MIP)/maximal expiratory pressure (MEP) or sniff nasal pressure (SNIP) if patients are symptomatic and FVC is > 50% predicted [34].

FVC is regarded as the standard indicator of ALS disease progression, however, studies have suggested that other pulmonary function measures may be more sensitive indicators of respiratory dysfunction [35]. A study reported that maximal inspiratory pressure (MIP) and maximal expiratory pressure (MEP) change fastest from the baseline in PwALS compared to the other pulmonary function measures, suggesting a more rapid deterioration of expiratory muscle strength [35]. A study [36] identified that Sniff Nasal Inspiratory Pressure (SNIP) and maximal inspiratory and expiratory pressures (MIP, MEP) are decreased earlier in the course of the disease and plunged deeper before death than VC. It is well known that breathing and swallowing dysfunction determine the prognosis of ALS [37]. Mechanistically, it has also been shown that the pressure of cricopharyngeus has a significant positive correlation with FEV1 in PwALS [20]. Therefore, the parameters measured in patients with ALS, such as the pressure of cricopharyngeus, could also be used to predict respiratory function [20], especially in patients who cannot conduct the spirometer due to weakness of orofacial and respiratory muscles [20].

The progression of dysphagia and its relationship with speech, articulation, and respiratory function is important for the timing of PEG placement [15]. Strand et al., reported that the progression of dysphagia follows a rate similar to that of speech and that functional disability resulting from bulbar deficits will affect speech and swallowing to a similar degree [15]. Mariani et al., reported that patients with bulbar onset have a shorter time from the beginning of symptoms to the onset of dysphagia symptoms, on the other hand, patients with Spinal Onset (SO) already had a swallowing disorder present in 52.5% of cases [19]. Patients with Bulbar Onset (BO) and fast progressing disease had a higher percentage (92.5%) of patients with swallowing already compromised at the first laryngological evaluation [19]. However, most (85%) did not experience aspiration at FEES, suggesting preliminary impairment only during the oral phase of swallowing with the preservation of laryngeal sensitivity [19]. That might mean that the evaluation of swallowing should not only include FEES but also other methods such as bedside clinical swallowing evaluation so that other swallowing impairments could be detected in addition to aspiration.

BMI can be a predictor of overall survival and specific survival after PEG placement [38]. Specifically, the difference of BMI between diagnosis and PEG insertion may indicate that PEG insertion should be planned earlier than currently recommended [18, 38]. It has been reported that there is an increase in survival in patients who did not present weight loss in the six months following PEG placement [18].

Patients’ priorities and concerns about PEG are also important to be taken into consideration. While most patients eventually consent to PEG insertion, this decision can take an extended period of time [9] regardless of the presence of swallowing impairment or nutritional compromise [23]. Labra et al. reported that the median time between medical discussion and PEG referral was < 1 week. Although, in almost 10% of the cohort the gap was > 1 month, with the longest taking 57 days. These findings highlight the combination of decisional conflict for some patients, as well as lengthy hospital waiting times to undergo the insertion procedure [23]. There is often disparity between patients’ decisions and practice guidelines, even when conditions for decision-making are optimal [39].

Yet, PwALS with both BO and SO experience swallowing difficulties over the course of the disease, with progressive deterioration. It has been argued that traditional stratification used for clinical trials with PwALS, namely BO and SO ALS is no longer sufficient or adequate [40]. On the other hand, it has been proposed that disease progression in relation to dysphagia symptoms progression may be more effective and ideal for intervention and decision-making for PEG placement [19].

This systematic review highlights the importance of swallowing assessment in the decision-making process for PEG insertion in PwALS. In spite of increasing clinical interest in dysphagia in PwALS, discrepancies related to its assessment and management are still evident and the assessment of swallowing in PwALS and their sensitivities and specificities should be studied further. A multi-factorial assessment of dysphagia as well as a multi-layered decision-making process that is tailored to the patient must be adopted for optimal timing of PEG insertion in PwALS.

## Conclusion

The decision-making process for PEG insertion is not clearly determined by clinical guidelines. Despite the heterogeneity of the PEG insertion criteria, dysphagia is a common criterion for the decision-making process. Even though there are different tools and assessments to judge swallowing deterioration, there is a need to decide on the optimal method that will assist decision-making effectively for PEG insertion. Early involvement of SLTs could facilitate improved decision-making for optimal timing for PEG insertion and support focused treatment strategies to maintain function and prevent complications associated with the disease.

## Supplementary Information

Below is the link to the electronic supplementary material.

Supplementary file1 (DOCX 14 KB)

## Data Availability

All data supporting the findings of this study are available within the paper and it’s Supplementary Information.
