# Supplementary material for: Dysphagia Assessments as Criteria in the ‘Decision-Making Process’ for Percutaneous Endoscopic Gastrostomy Placement in People with Amyotrophic Lateral Sclerosis: A Systematic Review
Source: Dysphagia. 2024 Mar 22;39(6):977–88. doi: 10.1007/s00455-024-10686-2 (PMC11606986; doi:10.1007/s00455-024-10686-2)
Supplement: Supplementary file 1 — Supplementary file1 (DOCX 14 KB) [file 455_2024_10686_MOESM1_ESM.docx]

**Supplemental material**

Table S1. Search terms

| PUBMED | ((((((amyotrophic lateral sclerosis[MeSH Terms]) OR (ALS[MeSH Terms])) OR (MND[MeSH Terms])) OR (motor neuron disease[MeSH Terms])) AND (((((((((dysphagia[MeSH Terms]) OR (swallowing disorder[MeSH Terms])) OR (swallowing disorders[MeSH Terms])) OR (deglutition disorder[MeSH Terms])) OR (deglutition disorders[MeSH Terms])) OR (eating disorders[MeSH Terms])) OR (feeding disorders[MeSH Terms])) OR (eating and feeding disorders[MeSH Terms])) OR (deglut disorders[MeSH Terms]))) AND (((((assessment[MeSH Terms]) OR (test[MeSH Terms])) OR (evaluation[MeSH Terms])) OR (diagnosis[MeSH Terms])) OR (screening tools[MeSH Terms]))) AND (((((PEG[MeSH Terms]) OR (Enteral tube feeding[MeSH Terms])) OR (percutaneous endoscopic gastrostomy[MeSH Terms])) OR (tube feeding[MeSH Terms])) OR (gastrostomy[MeSH Terms])) |
| --- | --- |
| Embase | Swallowing/ or Dysphagia/ or Feeding/ or Eating/ or Eating disorders/  AND  diagnos*/ or diagnosis/ or diagnostic*/ or diagnosis, differential/ or diagnosis/ or screening/ or asses*/ or protocol/ or [test.mp](http://test.mp/). [mp=title, abstract, heading word, drug trade name, original title, device manufacturer, drug manufacturer, device trade name, keyword heading word, floating subheading word, candidate term word]/  AND  Lou Gehrig*/ or Amyotrophic Lateral Sclerosis/ or Motor Neuron Disease/ or ALS/ or MND/  AND  enteral nutrition/ or nutritional support/ or percutaneous feeding.mp. or artificial feeding.mp. or artificial hydration.mp. or endoscopic gastrostomy.mp. or tube feeding.mp. or peg.mp. or enteral feeding.mp. or stomach tube$.mp. or forced feeding.mp. or percutaneous feeding.mp. or artificial nutrition.mp. or nutritional support.mp. or enteral nutrition.mp. or feeding methods.mp or tube$.mp. |
| CINAHL Plus | (((MH "Deglutition") OR (MH"Deglutition Disorders") OR (MH"Feeding of Disabled") OR (MH"Feeding Self Care Deficit (NANDA)") OR (MH "Feeding Methods") OR (MH"Eating Behavior") OR (MH "Eating Disorders Management (Iowa NIC)")OR (MH "Eating Disorders") OR (MH"Swallowing Impairment"))  AND  TX diagnosis OR TX screening ORTX protocol OR TX (assessment tools or assessment method or assessing or assessment or assessment strategies ) OR TX ( (Sensitivity and specificity ) OR TXROC curve )  AND  TX ( enteral nutrition OR nutritional support ) OR TX tube$ OR TX percutaneous feeding OR TX (artificial feeding OR forced feeding OR percutaneous feeding OR artificial nutrition ) OR TX feeding methods ORTX enteral nutrition OR TX artificial hydration OR TX endoscopic gastrostomy OR TX tube feeding OR TX peg OR TX enteral feeding OR TX stomach tube$  AND  TX ( amyotrophic lateral sclerosis orals or lou gehrig's disease ) OR TX (mnd or motor neuron disease ) |
